# Gender-related facilitators and barriers in implementing combined lifestyle interventions for individuals with knee osteoarthritis and overweight or obesity: perspectives of healthcare professionals and patients in a qualitative study

**DOI:** 10.1136/bmjopen-2025-112032

**Published:** 2026-05-12

**Authors:** Chantal M Hulshof, Nuria EJ Jansen, Priya Gharbaran, Dieuwke Schiphof, Pauwke PL Berkers, Marienke van Middelkoop

**Affiliations:** 1 Department of General Practice, Erasmus MC University Medical Center Rotterdam, Rotterdam, Netherlands; 2 Department of Arts and Culture Studies, Erasmus University Rotterdam, Rotterdam, Netherlands

**Keywords:** General Practice, Primary Health Care, QUALITATIVE RESEARCH, Overweight

## Abstract

**Abstract:**

**Objectives:**

Weight loss through combined lifestyle interventions (CLIs) can slow down knee osteoarthritis (OA) progression. However, gender-related factors may influence both the implementation of and participation in CLIs. Therefore, the aims were to identify gender-related facilitators and barriers to CLI implementation among healthcare professionals (HCPs) and uptake and adherence among individuals with early-stage knee OA and overweight or obesity.

**Design:**

Semi-structured interviews were conducted in this qualitative study. Thematic analysis, combining inductive and deductive approaches, was performed according to the Consolidated Framework for Implementation Research (CFIR) domains.

**Setting:**

HCPs and individuals with knee OA and overweight or obesity were interviewed in Dutch between December 2023 and May 2024.

**Participants:**

16 HCPs who referred or delivered a CLI and 23 individuals with knee OA and overweight or obesity who participated in a CLI were purposively recruited. 16 participants identified themselves as women and 7 as men; no other gender identities were reported.

**Results:**

HCPs experienced resistance to CLIs of both men and women. Additionally, HCPs perceived that women were more open and available to participate in CLIs, tended to prioritise others over themselves and often lacked familial support compared with men. Women valued trust, preferred female HCPs and struggled with self-prioritisation. Men were motivated by female HCPs and participants due to their empathy and interaction. Across men and women, barriers included conflicting HCP advice, work stress, caregiving tasks and lack of discipline, while social environment support was a key facilitator.

**Conclusions:**

Addressing gender-related facilitators and barriers in the design and implementation of the CLI may improve long-term engagement and efficacy in individuals with knee OA and overweight or obesity.

**Trial registration number:**

Overview of Medical Research in the Netherlands (OMON), managed by the CCMO: NL75367.078.20

**STRENGTHS AND LIMITATIONS OF THIS STUDY**

- The perspectives of both healthcare professionals and individuals with knee osteoarthritis and overweight or obesity involved in a combined lifestyle intervention were identified.
- Purposive sampling was used to include a diverse sample in terms of age, sex, socioeconomic status and attendance status to a combined lifestyle intervention.
- Only Dutch people were interviewed and diversity in gender and health literacy was limited, limiting generalisability.
- The risk of recall bias was minimised by conducting interviews during or shortly after participation in a combined lifestyle intervention.

## Introduction

 Knee osteoarthritis (OA) is a chronic and progressive joint disorder, leading to pain, stiffness and reduced physical function.^1^ It is one of the leading causes of disability worldwide, especially among older adults.^2^ In the Netherlands, OA is expected to become the most prevalent chronic disease by 2040, mainly due to the ageing population and the increasing prevalence of overweight and obesity.^3^ A high body mass index (BMI) is not only a major risk factor for the development and progression of knee OA, but also the most important modifiable factor in the treatment of knee OA.^4^ Therefore, early interventions aimed at body weight loss, such as lifestyle interventions, are essential to slow the progression of knee OA, improve quality of life and reduce pressure on healthcare systems.^5^

Combined lifestyle interventions (CLIs) have shown promise for individuals with knee OA who are overweight and obese.^6^ These interventions integrate physical activity, healthy nutrition and behavioural change strategies to support body weight loss and a healthier lifestyle.^7^ However, their effectiveness depends not only on clinical outcomes but also on successful referral and implementation by healthcare professionals (HCPs), as well as acceptance and adherence by individuals with knee OA. In primary care, HCPs such as general practitioners (GPs) and physical therapists recognise the importance of lifestyle interventions, but reported practical and organisational challenges.^8 9^ Furthermore, people’s individual circumstances, including gender-related social roles, responsibilities and expectations, determine whether a programme meets their needs and whether they can benefit from it.^10^

Gender-related factors may influence the implementation, uptake and adherence to CLIs in individuals with knee OA and overweight or obesity.^11^ HCPs may have unconscious biases regarding sex and gender,^12^ which can influence healthcare given to patients.^13 14^ Hence, sex and gender analyses can help to reveal such inequalities and develop strategies to promote more equitable healthcare.^11^,^13^ It is therefore essential to evaluate how HCPs perceive and interact with people of different genders,^15^ as these may influence referral patterns, engagement of individuals and the likelihood of adherence to a CLI. Current insights into this gender-related interaction in the CLI are limited, while understanding it can strengthen the patient-HCP relationship, supporting accessibility and long-term adherence to a CLI.

Although the CLI seems to meet the needs of individuals with overweight and obesity,^16^ men and women might differ in their preferences and motivations for a healthy lifestyle. Previous research has shown sex differences in motivations, with men more likely to be motivated by performance and fitness goals, while women are more likely to be motivated by weight management and improving well-being and health.^17^ Additionally, men often perceive dieting as feminine.^18 19^ Although both physical activity and healthy nutrition are integrated in CLIs, in practice the emphasis seems to be more on healthy nutrition. This imbalance is reflected in the underrepresentation of men in lifestyle trials and the fact that approximately 70% of CLI participants in the Netherlands are women.^20 21^ Difference in preferences, motivations, perceptions and experiences in the CLI can all influence both participation in and adherence to a CLI. However, the focus has mainly been on the content of lifestyle interventions, while differences in individual circumstances, motivations and communication preferences need to be further investigated.

Gaining insights into gender-related facilitators and barriers from multiple perspectives facilitates the implementation and acceptance of the CLI in both men and women. Therefore, this study aims to identify gender-related facilitators and barriers to the implementation of CLIs for HCPs who referred or delivered them. To complement the findings of HCPs, gender-related facilitators and barriers to the uptake and adherence of CLIs were investigated in individuals with early-stage knee OA and overweight or obesity.

## Methods

This study was reported according to the Standards for Reporting Qualitative Research and Sex and Gender Equity in Research.^22 23^ This qualitative study is an extension of the interview study conducted as part of the Lifestyle Intervention Trial for Early-stage knee OA (LITE) study.^24^ The LITE study was a multi-centre pragmatic randomised controlled trial in which participants in the intervention group followed the 2-year ‘Beweegkuur’ CLI.^25^ A total of 218 individuals participated in the LITE study, of whom 109 were allocated to the CLI. Participants in the LITE study were between 45 and 70 years of age, clinically diagnosed knee OA according to the National Institute for Health and Care Excellence (NICE) guideline^26^ had a BMI of ≥25 kg/m^2^ or higher, and consulted their GP for the first time with knee complaints in the previous 24 months.

We defined sex as ‘a set of biological attributes of humans, including physical features, chromosomes, gene expression, hormones and anatomy” and gender as “the socially constructed roles, behaviours, expressions and identities of women, men, and gender-diverse people’.^23^

### Study design

The design of this study is qualitative, following an interpretivist approach, to gain insights into perspectives of HCPs who referred or delivered a CLI and individuals with knee OA and overweight or obesity who participated in a CLI. Throughout the methods and results section of this manuscript, ‘HCPs’ and ‘participants’ refer to these respective groups. Semi-structured interviews were conducted with HCPs to identify gender-related barriers and facilitators for the design and implementation of the lifestyle intervention, and with individuals with knee OA to identify gender-related barriers and facilitators to the uptake and adherence of the CLI.

The interview guides and coding trees were guided by the updated Consolidated Framework for Implementation Research (CFIR).^27^ This framework is well-recognised in the implementation science field. The updated CFIR consists of five domains: innovation, outer setting, inner setting, individuals and implementation process. For this study, the interview guides were expanded with additional topics that specifically addressed gender-related factors. For HCPs, the additional topics were patient factors (eg, gender identity of patient, psychosocial), provider factors (eg, gender identity of HCP) and provider-patient relationship (eg, communication and expectations). For participants, the additional topics were healthcare-related factors (eg, gender identity of HCP, relationship and communication with HCP, expectation of HCP) and environmental factors (eg, social network, work, household activities and caretaking).

### Recruitment and sampling

HCPs (mix of GPs, lifestyle coaches and exercise professionals) who were involved in the LITE study and referred or provided care to at least two LITE participants were invited for the interviews. In addition, LITE participants allocated to the intervention arm were invited to participate in the interviews. Purposive sampling was used to recruit GPs and participants across different general practices, with a range of ages, sexes and number of CLI sessions attended (eg, LITE participants who have completed the CLI or dropped out early).

Furthermore, to gain insights into experiences with different CLI programmes, interviews were conducted with HCPs and participants who were not involved in the LITE study but were participating in CLI programmes other than the ‘Beweegkuur’. These participants were recruited via LinkedIn and the research team’s network.

### Data collection

Prior to the start of the interview, written informed consent was obtained from HCPs and participants. Between December 2023 and May 2024, the semi-structured interviews were conducted in Dutch, either online or in person at the department of General Practice in Erasmus MC when online participation was not possible. Two members of the research team (NEJ and PG) conducted the interviews with the HCPs and participants. Neither of them had a clinical relationship with the participants, and one of them (NEJ) knew the participants from the research visits. After the first interview, minor adaptions in wording of questions were made to clarify the questions in the interview guide. To ensure methodological consistency, the first two interviews were conducted by both researchers together. The remaining interviews were conducted by one researcher. Prompts were provided to HCPs and participants when they had difficulties with giving their own examples. The interviews lasted approximately 45–60 min. All were video recorded via Microsoft Teams if conducted online or audio recorded via a voice recorder if conducted in person. All interviews were transcribed verbatim. Data saturation was considered reached when new interviews did not give any additional insights.

Demographic data were collected at the beginning of the interview from HCPs and via an online questionnaire from participants as standard procedure of the LITE study. Sex and gender identity were self-reported using the following questions, translated from Dutch: ‘What was your sex at birth?’ and ‘What is your gender identity?’, respectively. In addition, gender-related data were collected exclusively from participants involved in the LITE study (n=218) using the Dutch version of the Stanford Gender-related Variable for Health Research questionnaire.^28 29^ These data were not obtained from participants who were not involved in the LITE study and HCPs. This questionnaire contains questions on seven gender-related variables: work strain, social support, risk taking, independence, emotional intelligence, discrimination and caregiver strain.

### Data analysis

Thematic analysis, combining inductive and deductive approaches, was performed using MAXQDA 2018 (VERBI Software GmbH, Berlin, Germany). First, two researchers (NEJ and PG) independently coded two interview transcripts inductively to develop initial codes. Then, the codes were connected to one of the five CFIR domains and organised into separate coding trees for HCPs and participants reflecting both perspectives. Following a discussion with a third researcher (DS) about the coding trees, the coding trees were revised, and another interview transcript was coded using the revised coding trees. After 75% inter-rater reliability was achieved between two researchers (NEJ and PG), based on code definition and overlap in themes, one researcher (PG) coded the remaining transcripts.

For the purpose of this study, we made separate coding trees for each gender identity. If demographic data on self-reported gender identity were missing, we assumed the gender identity of participants on the unspecified question ‘What is your sex/gender’ at the baseline of the LITE study. Themes and subthemes were created based on related codes, and discrepancies were resolved through a consensus meeting with the research team (CMH, NEJ, PG, DS, PPLB and MvM). For HCPs, we have only focused on the outer setting domain of CFIR, specifically on the influence of gender. For participants, we focused on the domains of outer setting, inner setting and individuals from CFIR. The themes and subthemes were linked to the above-mentioned domains. Quotes presented for each subtheme are translated from Dutch to English.

Credibility was ensured through triangulation, as we involved both HCPs and CLI participants to capture diverse perspectives. Dependability was supported by a systematic coding process and assessment of inter-rater reliability. Coding decisions and thematic interpretations were refined through discussions within the researcher team. Confirmability was ensured by linking the themes and subthemes to the CFIR framework. The quotes further demonstrate that the findings are supported by the data.

### Patient and public involvement

Patient involvement was promoted via the Artrose Gezond (ie, Healthy with OA) platform, which is coordinated by Erasmus MC. Input from patients and HCPs was incorporated into the development of interview guides. In addition, patients contributed to the research design and interpretation of the data through workshops. The findings were also discussed with GPs and lifestyle coaches to gather professional insights and explore the implications for clinical practice.

## Results

In total, 16 HCPs and 23 individuals with knee OA and overweight or obesity were interviewed. HCPs were a mix of GPs (n=7), lifestyle coaches (n=8) and an exercise professional (n=1), identified themselves as women (50%) or men (25%) (25% missing) and had a median age of 39 years (range: 31–59). Participants identified themselves as women (70%) or men (30%), were mostly adequately health literate, had a mix of socioeconomic statuses, a median age of 56 years (range: 46–71) and 87% participated in the ‘Beweegkuur’ CLI and 13% in another CLI (table 1). As no HCPs and participants identified themselves as non-binary or genderqueer, the results were limited to the gender identities of man and woman.

**Table 1:** Characteristics of individuals with knee OA and overweight or obesity

* *	All (n=23)	Women (n=16)^*^	Men (n=7)^*^
Sex			
Female	16 (70%)	16 (100%)	0 (0%)
Male	7 (30%)	0 (0%)	7 (100%)
Age (years)	56 (range: 46–71)	56 (range: 47–67)	58 (range: 46–71)
Socioeconomic status^†^			
Low** **	7 (30%)	4 (24%)	3 (43%)
Middle	9 (39%)	7 (44%)	2 (29%)
High	7 (30%)	5 (31%)	2 (29%)
Health literacy‡			
Adequate	17 (74%)	12 (75%)	5 (71%)
Marginal	2 (9%)	1 (6%)	1 (14%)
Inadequate	0 (0%)	0 (0%)	0 (0%)
Missing	4 (17%)	3 (19%)	1 (14%)
Attendance status CLI			
Completed	12 (52%)	9 (56%)	3 (43%)
Participating	6 (26%)	5 (31%)	1 (14%)
Stopped early	5 (22%)	2 (13%)	3 (43%)
CLI programme			
Beweegkuur (LITE study)	20 (87%)	14 (87%)	6 (86%)
Other	3 (13%)	2 (13%)	1 (14%)

Note: Discrete data are number (percentage) and continuous data are median (range).

* Four gender identities were missing

† Socioeconomic status determined by the highest level of education attained.

‡ Health literacy assessed using Brief Health Literacy Screening Tool (BRIEF): Inadequate Health Literacy (3–8), Marginal Health Literacy (9–11) and Adequate Health Literacy (12–15).

CLI, combined lifestyle interventions; LITE, Lifestyle Intervention Trial for Early-stage knee OA; OA, osteoarthritis.

The definitions of the identified subthemes in the outer setting domain of CFIR within the theme ‘Influence of gender’ are provided in table 2. Quotes supporting the findings regarding the facilitators and barriers are presented separately for men and women for each subtheme in online supplemental table S1 (Q… references to the corresponding quotes (HCP, healthcare professional; W, woman; M, man).

**Table 2:** CFIR domain, theme and subthemes for the implementation of the CLI

CFIR domain and theme	Subthemes	Definition
Outer setting: influence of gender	Participant–HCP relationship	The trust, communication and interaction between participant and HCP in the CLI.
	Work-life balance	Balance between professional and personal life to enable participation in the CLI.
	Behavioural factors	Factors that influence behaviour in the CLI.
	Social environment support	Assistance and comfort from friends, family and colleagues to participate in the CLI.

CFIR, consolidated framework for implementation research; CLI, combined lifestyle intervention; HCP, healthcare professional.

### Subtheme: participant–HCP relationship

HCPs perceived that women tend to be more social, open and sincere (Q1, HCP13) and are more likely to follow the CLI than men (Q2, HCP04). In general, HCPs observed larger participation of women in the CLI. Participants who identified themselves as woman mentioned that changes in HCPs or no match with HCPs may hinder the openness, because they need to rebuild trust with an HCP (Q3, W07 and Q4, W12). Furthermore, some women prefer to discuss topics such as weight loss and menopause with a female HCP (Q5, W03), but more than half of them have no preference for the gender of an HCP. HCPs experienced that men are less open and find it challenging to share personal information and their true feelings (Q6, HCP11 and Q7, HCP13). Based on individual experiences, one participant identified as a man observed that the presence of women in CLI group sessions led to more interaction, and another man appreciated the empathy that female HCPs often showed (Q8, M06). Most men have no preference for the gender of an HCP.

Regarding communication, both men and women experienced that the progress of the CLI can be hampered by different opinions of HCPs (Q9, W05), poor communication or failure of HCPs to keep appointments (Q10, M04).

### Subtheme: work-life balance

**Figure 1:**
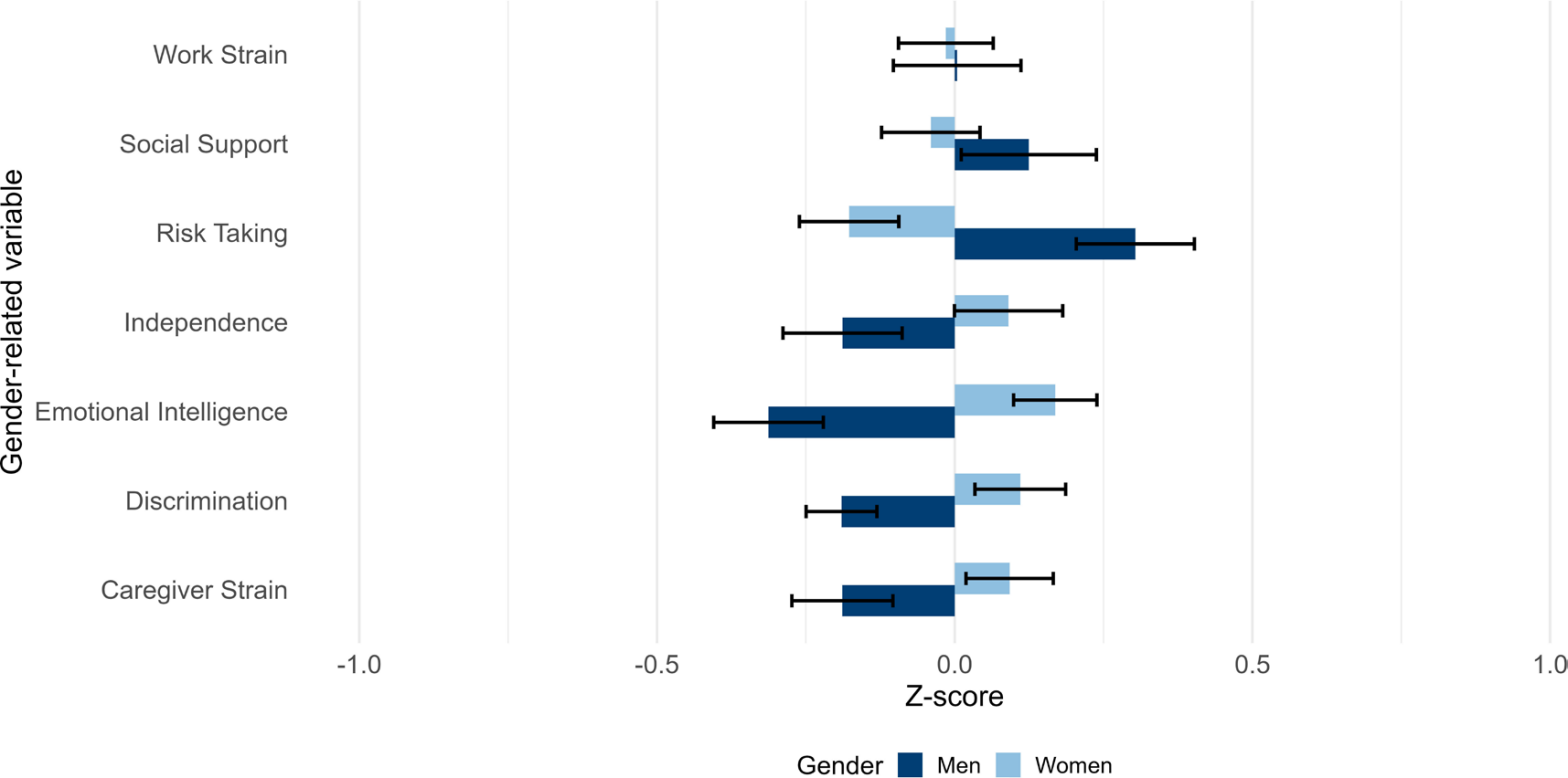
Responses to the Stanford Gender-related Variable for Health Research (GVHR) questionnaire from 163 LITE participants who identified themselves as man or woman. LITE, Lifestyle Intervention Trial for Early-stage knee.

HCPs mentioned that men have less time to participate in the CLI because they are more likely to work full-time than women (Q11, HCP08 and Q12, HCP16). Both participants who identified themselves as man or woman experienced that the CLI could be followed alongside work (Q13, W06 and Q14, M06). On the other hand, both perceived irregular working hours, stress or physical demands at work as a barrier to engaging in sports and changing their diet (Q15, W11 and Q16, M02). In addition, some women mentioned that scheduling appointments was sometimes challenging because they conflicted with working hours (Q17, W05). Responses to the Gender-related Variable for Health Research (GVHR) questionnaire on work strain align with the interview responses from both men and women regarding work-life balance, showing similarities in experiences of work strain between men and women (figure 1).

Most men and women did not seem to have traditional gender roles at home and seem to have an equal division of labour at home (Q18, M06). However, some did have traditional gender roles, with women mainly caring for their home and children while men worked more (Q19, W02). Regarding care responsibilities, some men and women cared for their grandchildren once or twice a week (Q20, M04). In addition, one man had care tasks for his partner because she had health problems. Some women were informal carers for their parents (Q21, W10), and one woman had to care for her child with an intellectual disability. Traditional gender roles and care responsibilities may affect the ability to participate in CLI programmes, due to time constraints and limited flexibility in managing daily responsibilities. Responses to the GVHR questionnaire on caregiver strain showed slightly higher levels of caregiver strain in women compared with men, although the difference was small (figure 1).

### Subtheme: behavioural factors

An HCP mentioned that men are more direct and less complex, for example, they just follow advice without asking why (Q22, HCP16), while women have more excuses and show more resistance to the CLI (Q23, HCP16). However, other HCPs perceived more resistance to the CLI in men than in women (Q24, HCP05). Both participants who identified themselves as man or woman perceived barriers of discipline. Both seem to know what they should do to eat healthier or start exercising but seem to fail to adjust their behaviour (Q25, M06, Q26, M03 and Q27, W09). Additionally, an HCP mentioned that women tend to seek alternative treatments, such as Saxenda, more quickly (Q28, HCP09).

HCPs mentioned that women often put themselves last because they have a lot on their minds and are busier with others (Q29, HCP16). This was experienced by women, facing stress due to both personal reasons and external factors, such as illnesses and deaths of family and friends, which sometimes even made them quit the CLI (Q30, W03).

### Subtheme: social environment support

HCPs noted that women sometimes lack social support from their partners and children, who are unwilling to adapt and therefore fail to create a supportive environment (Q31, HCP15). This was not specifically mentioned by participants who identified themselves as woman. Both men and women did mention that social support contributed to healthy eating habits, sports practice and participation in lifestyle interventions (Q32, M06 and Q33, W09). Men referred only to social support from their partners, while women additionally referred to social support from family and partners. Responses to the GVHR questionnaire on social support indicate almost comparable levels of perceived social support between men and women, with slightly higher scores in men (figure 1).

### No differences between men and women

Besides the differences mentioned by HCPs, six HCPs noted that they think that there is no difference between men and women in the implementation, uptake and adherence to the CLI. HCPs mentioned that it is very personal, and that age plays a role, with older people generally being more receptive to the CLI compared with younger people.

### GVHR questionnaire results

A total of 163 LITE participants completed the GVHR questionnaire. Figure 1 shows the gender-related variables stratified according to participants who identified themselves as man or woman. Men experienced higher work strain, had more social support and were more willing to take risks. Women valued independence more, had greater emotional intelligence, felt more discrimination and experienced higher caregiver strain. However, differences between genders were generally small.

## Discussion

The study aim was to identify gender-related facilitators and barriers to the implementation of CLIs for HCPs who referred or delivered a CLI. To complement the findings of HCPs, gender-related facilitators and barriers to the uptake and adherence of CLIs were identified in individuals with early-stage knee OA and overweight or obesity who participated in a CLI. Since none of the participants identified as non-binary or genderqueer, the results were limited to men and women. In general, HCPs perceived resistance to CLIs from participants who identified themselves as either man or woman. They perceived that women were more open and available to participate in CLIs, tended to prioritise others over themselves and often lacked familial support compared with men. While both men and women mentioned social support of their environment as a key facilitator. In addition, barriers for both men and women included conflicting HCP advice, work stress, caregiving tasks and lack of discipline. Furthermore, women valued trust, preferred female HCPs and struggled with self-prioritisation. Men were motivated by female HCPs and participants due to their empathy and interaction. There were also HCPs who mentioned no difference between men and women.

Individuals who identified themselves as woman with knee OA and overweight or obesity value a personal connection with their HCP, indicating a preference for emotional empathy—feeling another person’s emotions.^30 31^ Furthermore, HCPs perceived women as more social, open and likely to follow the CLI, while men were seen as more reserved, especially in sharing personal information. In line with the experience from HCPs and participants, emotional empathy is higher in women than in men.^30 31^ However, differences in cognitive empathy—understanding another person’s emotions—between men and women are less clear.^31^ Higher empathy in women is linked to more effective and empathetic communication styles, which can improve social interactions.^32^ Awareness among HCPs of individual differences in emotional empathy can help build stronger relationships with participants and may improve their uptake of and adherence to a CLI.

Although most interviewed participants did not mention traditional gender roles, some women did mention that their husbands generally worked more, while they themselves took on more responsibility for their children and household tasks. In addition, both men and women mentioned having informal caregiver tasks. Both traditional gender roles at home and informal caregiver tasks contribute to higher stress and emotional exhaustion,^33^ which may affect participation in a CLI. Daily conversations about work, household and care tasks can lead to a more equal and satisfying division of labour, which can reduce stress.^34^ An imbalance between work, caring and household tasks can lead to stress in both men and women, making it essential to address in CLIs. Therefore, understanding participants’ stressors such as their roles at home and their responsibilities as carers, and incorporating these into CLI sessions can improve engagement with a CLI.

HCPs perceived resistance from individuals who identified themselves as man or woman with knee OA and overweight or obesity. Participants, however, noted that although they intended to change their behaviour, they experienced difficulties in doing so. This gap between having the intention to change behaviour and actually changing behaviour is known as the intention-behaviour gap.^35^ The evidence for gender differences in the intention-behaviour gap remains inconclusive; some studies report that men are more likely to translate intentions into action, whereas other studies find the opposite pattern.^36^ Previous research suggests that perceived social support, intention, planning and self-regulation play key roles in bridging the intention-behaviour gap.^37^

Of these factors, social environment support was mentioned by both men and women in our study as a key facilitator in the uptake of healthier eating habits and participation in exercise and lifestyle interventions. HCPs observed a lack of social support among women, which is consistent with the findings of a previous qualitative study on adherence to a dietary lifestyle intervention in people with metabolic syndrome.^38^ However, individuals who identified themselves as women did not report a lack of social support. Men mentioned that they only received social support from their partners, while women also mentioned they received support from family and friends. This is in line with previous research showing that men mainly received non-work-related social support from their spouses, while women rely more on relatives and friends.^39^ Sources of social support appear to vary among individuals, but in general, social support is associated with positive health outcomes.^40^ It is therefore important that HCPs discuss the social support in participants’ environments and its implications during the CLI sessions.^41^

This qualitative study is the first to identify gender-related facilitators and barriers in the implementation, uptake and adherence to CLIs. This is relevant given the increasing recognition of gender differences in OA healthcare.^15^ A strength of this study was the inclusion of perspectives of both HCPs and individuals with knee OA and overweight or obesity involved in a CLI. Additionally, we included a diverse sample in terms of age, sex, socioeconomic status and CLI attendance status due to purposive sampling. However, a limitation was the generalisability because we only interviewed Dutch people and the diversity in gender identities and health literacy was limited. Furthermore, we included relatively older participants (median age 56 years), which may have influenced the diversity in gender identities and perspectives on gender, as generational differences often determine how individuals perceive and deal with these concepts.^42^ Future research would benefit from greater diversity in gender identities, health literacy and a broader age range to promote more inclusive and equitable healthcare that better meets the needs of patients. Another limitation is the lack of perspectives from individuals who did not participate in a CLI, which could provide insight into barriers to starting a CLI. Although interviews may introduce a risk of recall bias, conducting them during or shortly after CLI participation helped to minimise this risk.

HCPs and individuals with knee OA and overweight or obesity were mostly on the same page in terms of facilitators and barriers in individuals who identified themselves as either man or woman, but there were also some differences between their perceptions. Therefore, it is important not to stigmatise but to take an individual approach when referring or delivering a CLI. Furthermore, incorporating gender-related facilitators and barriers or at least addressing the subthemes found in the present study (ie. participant–HCP relationship, work-life balance, behavioural factors and social environment support) may improve the relationship between participant and HCP, potentially making a participant more willing to adhere to the CLI. Not all of these facilitators and barriers can be directly applied or modified within the design and implementation of CLIs. Nevertheless, they could potentially be addressed and incorporated into the content of CLI sessions. Whether these gender-related facilitators and barriers in CLIs can be addressed in the design or implementation of CLIs needs to be investigated in future implementation research. Subsequently, this may improve adherence to a CLI, which may result in a greater behavioural change among participants.

While the aim of a CLI is weight loss, the strategies to achieve this can vary. Previous research has shown gender differences in the motivational factors for physical activity. Women are more likely to be motivated by losing or managing weight and social factors (ie, together with peers and other women), while men are more likely to be motivated by skilled, vigorous and competitive physical activity.^43^ In addition, men and women differ in their health beliefs about which foods are considered healthy, which influences their dietary behaviour.^44 45^ Due to gender differences in motivations and health beliefs, individuals may benefit from different types of support during CLI participation. Therefore, future research should investigate whether incorporating these preferences into a CLI can improve adherence to and effectiveness of a CLI across different genders.

Gender-related facilitators and barriers to the implementation and acceptance of the CLI were identified. The design and implementation of the CLI could benefit from incorporating gender differences in communication and interaction preferences, motivation for physical activity and health-related dietary beliefs. In addition, incorporating insights into work-life balance and social environment support may improve its long-term efficacy in individuals with knee OA and overweight or obesity.

## Supplementary material

10.1136/bmjopen-2025-112032 online supplemental file 1

## Data Availability

Data are available upon reasonable request.
