# Supplementary material for: Gender-related facilitators and barriers in implementing combined lifestyle interventions for individuals with knee osteoarthritis and overweight or obesity: perspectives of healthcare professionals and patients in a qualitative study
Source: BMJ Open. 2026 May 12;16(5):e112032. doi: 10.1136/bmjopen-2025-112032 (PMC13182479; doi:10.1136/bmjopen-2025-112032)
Supplement: online supplemental file 1 [file bmjopen-16-5-s001.pdf]

Supplementary File: Table S1

Table S1 – Quotes supporting the findings regarding the facilitators and barriers are presented separately for men and women for each subtheme

	Women	Men
**Participant-HCP relationship**	*<u>Facilitator:</u> "With women, it's a bit easier. If they feel comfortable, they are also a bit more open and sincere. And then you can actually be more targeted."* ***(Q1, HCP13)***<br>*<u>Facilitator:</u> "Well, look, in my opinion women are generally, if you can put it that way these days, a bit more inclined to think in that direction. Look, in a general sense, I think women are generally a bit more inclined to go along with that (CLI)."* ***(Q2, HCP04)***<br>*<u>Barrier:</u> "Well, mainly that you first, first have to get used to each other. You expose a piece of your own life, why you, and you can tell that three or four times. And I had done that two or three times and then there was another one. I don't know exactly how many times it was, but that. And the last one was a really nice one, but also got sick, yes. Nothing to do about it. Sad for her, but also sad for me."* ***(Q3, W07 – Inner setting)***<br>*<u>Barrier:</u> "Well, I don't know, I don't think we had that click or anything. She sat back with her arms crossed from tell me. So, I didn't have a good feeling about that."* ***(Q4, W12 – Inner setting)***<br>*<u>Facilitator:</u> "I think as a woman to woman, especially with the personal stuff we have sometimes. I mean, I have absolutely no problem with male doctors, but*	*<u>Barrier:</u> "Yes well, men often find it harder to share things. That's one thing and especially vulnerable things."* ***(Q6, HCP11)***<br>*<u>Barrier:</u> "And naming too, right? So sometimes - they never name, or in the case of men (unintelligible), they don't name what is going on. They name other things. And then if you start naming things between the lines, they don't have to say it themselves. Then they can just say of yes, indeed, that's it. Or yes, exactly."* ***(Q7, HCP13)***<br>*<u>Facilitator:</u> "No, yes, maybe because I prefer talking to a female GP rather than a male one, but that is more of a slight personal preference because of the basic feeling. But an empathetic male GP is just as good as an empathetic female GP. Or whatever gender, none of that matters to me."* ***(Q8, M06 – Outer setting)***<br>*<u>Barrier:</u> "The physio, I went there once. And there I had a very nice, animated conversation with the best man, and he made all kinds of promises about sending sites and tools. I don't know what else. I never got that, so that was immediately done for me. I didn't go there again either. If this is how it works, then for me it doesn't have to. If I go to the physio for an appointment, I expect to have to do something there.*

	indeed I think it's the link with a woman about how you feel, how you feel in a body you're not completely happy with because you want to lose weight. For that, I still click more with someone of my own sex." ***(Q5, W03 – Outer setting)***<br>Barrier: *"... and what I have also noticed is that they (lifestyle coaches) all have different opinions about certain things, about what is good and what is not good. It's not clearly a line. So that was sometimes, that was annoying. I did point that out, also during the study, that that was difficult, and I think they did do something with those complaints towards the lifestyle practice."* ***(Q9, W05 – Inner setting)***	*But this was just a conversation about small talk and an appointment that was not kept, so I never actually went there again."* ***(Q10, M04 – Inner setting)***
**Work-life balance**	Facilitator: *"But men generally work more full-time than women, so that makes the difference. So, women have more time to do other things. Also to participate in group meetings or whatever, so I do think that makes a difference."* ***(Q11, HCP08)***<br>Facilitator: *"I work from home a lot. I can work in the evening. I decide my own working hours actually. They are very flexible in terms of work here."* ***(Q13, W06 – Outer setting)***<br>Barrier: *"Right, I think there is definitely a connection because I was also working irregular hours at times, but it was also physically very tough. I think to myself, oh I can do that, the physical part too (of the CLI), but I noticed with my knee and my back that it just really didn't work out."* ***(Q15, W11)***<br>Barrier: *"But those group sessions, that's really such a set time and yes, then you are really expected to be*	Barrier: *"And with men, I see that working quite often. Yes, there is a difference in that too, of course. It is not that they are all the same, but with men it is often the case that all the time goes into the work."* ***(Q12, HCP16)***<br>Facilitator: *"Fine (to combine CLI with work). My employer gives me space and I only work four days, so that also helps."* ***(Q14, M06 – Outer setting)***<br>Barrier: *"We actually cook too little, but that's also because of course I work irregularly and am sometimes late (from work) and then, then sometimes we eat too simple, but on weekends we try to pay more attention to that."* ***(Q16, M02 – Outer setting)***<br>Neutral factor: *"Well, that's actually kind of 50/50 (housework). Maybe I do a bit more, but it's fairly divided. We both made a list of things we dislike, and*

	there. Yes, that's not always possible (because of work).” ***(Q17, W05 – Outer setting)***<br>*Neutral factor:* *“Yes, when my husband comes from work, I have already cooked, because I work until 3.30pm. Then I'm already home for three quarters of an hour or an hour, because he works until 4.30pm. So, in those three quarters of an hour, I then cooked something very quickly. Then he comes home, and we can have dinner at five o'clock."* ***(Q19, W02 – Outer setting)***<br>*Neutral factor:* *"Yes, that's my mother. I'm quite busy with that. Not every day, but at least once a week I cook for her and go to see her. And phone every day, of course. I also go out with her, because she's quite, she does have a walker, but she still likes it when I'm there."* ***(Q21, W10 – Outer setting)***	*the other does these things.”* ***(Q18, M06 – Outer setting)***<br>*Neutral factor:* *“We have the grandchildren once every fortnight, whom we look after. They then stay over from Monday to Wednesday evening. And we do that together.”* ***(Q20, M04 – Outer setting)***
**Behavioural factors**	*Barrier:* *“With women, it is (motivation) very different. Women often know better. Or yes, they already have so much experience with losing weight, for example, that they start asking a lot of questions. Which of course is fine, but you often notice with women more excuses, resistance and with men, yes that's just less.”* ***(Q23, HCP16)***<br>*Barrier:* *"... making healthy choices is not that difficult. In my head I know it all, but in practice I do. Then sometimes it can be difficult, and I live alone and yes. Then sometimes you have to be on discipline, that you think no, I'm not going to order those fries. I'm going to cook now because I have all the stuff in the house. It doesn't happen often, but sometimes it does. Especially if you've been working hard or have a bit of*	*Facilitator:* *“Yes absolutely (difference in motivation), I find that I, but I always have that with men, men are also often more direct and simple-minded so to speak. So, they are often open to what I say and they often just do it, without asking questions. Or they're like, okay, well, I just have to do this.”* ***(Q22, HCP16)***<br>*Barrier:* *“And for the rest, I have more women who are a bit more into it anyway (the CLI). That men are somehow more resistant to it."* ***(Q24, HCP05)***<br>*Barrier:* *“Yes, that's true, but that helps, right? When you go to exercise, the hardest part of exercising is packing your bag. That's the hardest part of exercising."* ***(Q25, M06 – Individuals)***<br>*Barrier:* *“Yes. She said you can also set it (electric bike support) lower. Then I said yes that's right, but then I*

a dip, but that actually does happen." ***(Q27, W09 – Individuals)***

<u>*Barrier:*</u> *"I think women back then were more inclined to look at alternative methods. Not just lifestyle in general but immediately start talking about Saxenda for example. Indeed, I hadn't even noticed that until I got this question, but that is indeed the difference between men and women yes."* ***(Q28, HCP09)***

<u>*Barrier:*</u> *"Yes, with women you often see that the head of the family is too busy with other things. Actually, busy with everything but themselves. With men, it is often work that takes up all their time. Of course, there is a difference, but in many cases work dominates men's lives. With women, it is more about not taking time for themselves, not taking care of themselves. As long as others are well off, that's what it's about. You see that a lot, yes."* ***(Q29, HCP16)***

<u>*Barrier:*</u> *"I was doing that (sports) with my very best friend, and she was suddenly diagnosed with extensive lung cancer and then you notice that your support goes the other way. You start supporting her and helping her, and indeed with myself going on with how I felt, then I also broke my ankle which I still suffer from, yes then. As for exercising, where I normally had to really discipline myself to get in the car to exercise, now the gym is five minutes away and I'm a nine-month sponsor now and I think I've been there six times in those nine months. I also need to start kicking myself again. That discipline of come on, those 20 kilos have to come off again, which was added during*

won't go as fast. And somewhere I do want to be more flexible. Not that I have to pedal really hard and sweat a bit. That's nice for when you're going home, but not when you're going somewhere. So, we had some discussions about that. But yes, the motivation." ***(Q26, M03 – Individuals)***

	*the COVID time. And that's tough." **(Q30, W03 – Individuals)***	
**Social environment support**	<u>*Barrier:*</u> *"What does often happen, I have noticed, is that there are some women who want to change, but the home situation does not cooperate. So, the husband doesn't want to eat healthy, or the child, or whatever, and then you find that, very often in the CLI, people participate and want to change things, but not everyone in the household is involved, so to speak. That also makes it very difficult." **(Q31, HCP15)***<br><u>*Facilitator:*</u> *"Yes and then I can imagine it in a group, which is stimulating anyway. I also train with two girlfriends. Every Monday strength training and because you have that appointment you go. And yes, and otherwise if the weather is bad, you think oh you know what, never mind." **(Q33, W09 – Outer setting)***	<u>*Facilitator:*</u> *"Well, by exercising together (with partner) and paying close attention to our diet together. So, she already had the dietician and putting that side by side. How can we combine that? And just thinking about that together. We always sit down together once a week and make a shopping list. What are we going to eat? And then I get to do the shopping on Tuesdays. And making sure we are indeed outside a lot, just moving around, especially walking, that helps. We enjoy that ourselves." **(Q32, M06 – Outer setting)***

Note: Q = quote, HCP = healthcare professional, W = women, M = men, GP = general practitioner.
