# Supplementary material for: Gender-related facilitators and barriers in implementing combined lifestyle interventions for individuals with knee osteoarthritis and overweight or obesity: perspectives of healthcare professionals and patients in a qualitative study
Source: BMJ Open. 2026 May 12;16(5):e112032. doi: 10.1136/bmjopen-2025-112032 (PMC13182479; doi:10.1136/bmjopen-2025-112032)
Supplement: Supplementary file 2 [file bmjopen-2025-112032.reviewer_comments.pdf]

PEER REVIEW HISTORY

BMJ Open publishes all reviews undertaken for accepted manuscripts. Reviewers are asked to complete a checklist review form and are provided with free text boxes to elaborate on their assessment. These free text comments are reproduced below.

ARTICLE DETAILS

Title (Provisional)

Gender-related facilitators and barriers in implementing combined lifestyle interventions for individuals with knee osteoarthritis and overweight or obesity: perspectives of healthcare professionals and patients in a qualitative study

Authors

Hulshof, Chantal M; Jansen, Nuria E; Gharbaran, Priya; Schiphof, Dieuwke; Berkers, Pauwke PL; van Middelkoop, Marienke

---

VERSION 1 - REVIEW

**Reviewer**	**1**
**Name**	**Zwar, Nicholas**
**Affiliation**	**Bond University, Health Sciences & Medicine**
**Date**	**30-Nov-2025**
**COI**	**None**

This qualitive study seeks to explore perceptions of health professionals on gender-related facilitators and barriers to Combined Lifestyle Intervention (CLI) implementation and perception on gender-related influences on uptake and adherence to a CLI among individuals with early-stage knee OA and overweight or obesity.

The study has a range of limitations, some of which are identified by the authors. These include it was conducted in only one country and many of the participants were from a study of a particular CLI (LITE study). It is not clear to what extent other people with knee OA were involved and importantly the extent to which the views of those who had not taken up the opportunity of participating in a CLI were included.

The project sets out to identify and address gender-related facilitators and barriers in the design and implementation of a CLI in order to improve long-term engagement and efficacy in individuals with knee OA and overweight or obesity. However, the majority of issues identified such as gender of the HCP referring to or providing the CLI, work stress, care-giving responsibilities, extent of social support among participants are largely not factors the design and implementation of the CLI can address.

There does not seem to have been exploration in the interviews on whether either HCPs and/or CLI participants thought gender barriers could be addressed in CLI design and implementation.

Reviewer **2**

Name **Ji, Hong**

Affiliation **Nursing department, The First Affiliated Hospital, Shandong First Medical University**

Date **16-Jan-2026**

COI **None**

1.The Introduction section lacks sufficient logical coherence.

2.The description of the credibility test in qualitative analysis is not detailed enough. It fails to explain the specific process, judgment criteria, and dispute resolution mechanism of the consistency test.

3.The classification system needs further optimization and adjustment.The logic of the affiliation of some sub-topics is unreasonable.For example, some content related to "Social Support" is scattered across different themes, and the classification of some subdivisions related to "Motivation" is not clear enough.

4.The Discussion section is underdeveloped and lacks sufficient depth. The interpretation of research results is superficial, failing to fully conduct comparative analysis with existing relevant studies. The exploration of underlying mechanisms such as gender culture and social structure behind the results is insufficient, and the elaboration on the clinical transformation value of the results is not specific enough.

5.Deepen the reflection on the limitations of the research and provide suggestions for future research improvements.

VERSION 1 - AUTHOR RESPONSE

Reviewer: 1
Prof. Nicholas Zwar, Bond University

Comments to the Author:

Comment 1: *This qualitive study seeks to explore perceptions of health professionals on gender-related facilitators and barriers to Combined Lifestyle Intervention (CLI) implementation and perception on gender-related influences on uptake and adherence to a CLI among individuals with early-stage knee OA and overweight or obesity.*

Response: Thank you for your time and thoughtful review, we truly appreciate it.

Comment 2: *The study has a range of limitations, some of which are identified by the authors. These include it was conducted in only one country and many of the participants were from a study of a particular CLI (LITE study). It is not clear to what extent other people with knee OA were involved and importantly the extent to which the views of those who had not taken up the opportunity of participating in a CLI were included.*

Response: Thank you for your comment. We appreciate your careful consideration of the limitations of the study. In response, we have added whether participants took part in the 'Beweegkuur' CLI or another CLI in the manuscript text and in Table 1, in addition to reporting the attendance status of the CLI (i.e. completed, participating, stopped early). Table 1 was formerly Table 2; the original Table 1 has been removed in accordance with comment 3 of the Editor. Additionally, we have now added a limitation noting the lack of perspectives from individuals who did not participate in a CLI. Although this study was designed to investigate the experiences of healthcare professionals and participants within a CLI, the views of non-participants could also have provided insight into barriers to starting a CLI.

Action: The following has been added in the text
Page 9, lines 262-265, Table 1:

> Participants identified themselves as woman (70%) or man (30%), were mostly adequately health literate, had a mix of socioeconomic statuses, and a median age of 56 years (range: 46-71) and 87% participated in the 'Beweegkuur' CLI and 13% in another CLI (Table 1).

Page 22, lines 445-447:

> Another limitation is the lack of perspectives from individuals who did not participate in a CLI, which could provide insight into barriers to starting a CLI.

Comment 3: *The project sets out to identify and address gender-related facilitators and barriers in the design and implementation of a CLI in order to improve long-term engagement and efficacy in individuals with knee OA and overweight or obesity. However, the majority of issues identified such as gender of the HCP referring to or providing the CLI, work stress, care-giving responsibilities, extent of social support among participants are largely not factors the design and implementation of the CLI can address.*

Response: Thank you for your comment. You are correct that several gender-related barriers identified in our findings, such as work stress, care responsibilities, social support, and the gender of the referring or treating healthcare professionals, go beyond what can be directly influenced by the design or implementation of a CLI. However, we were not only interested in the facilitators and barriers to the design and implementation of the CLI, but also in facilitators and barriers to the uptake and adherence of CLIs among participants with early-stage knee OA and overweight, as mentioned in our aim. We therefore consider these factors highly relevant, because

they determine the broader context in which individuals accept the CLI and because they influence their ability to adhere to the CLI. Although these factors cannot be fully resolved within the design or implementation of the CLI, it is important that they are recognised and integrated into the content and discussions during CLI sessions. By addressing these issues, healthcare professionals can better anticipate challenges, tailor their communication, and offer strategies that are relevant to the challenges individuals face in their daily lives. In this way, the CLI can respond better to gender-specific needs and create a supportive environment that increases engagement and adherence. We have refined this in the discussion.

Action: The following has been refined in the discussion :
Page 22, lines 454-461:

> Furthermore, incorporating gender-related facilitators and barriers or at least addressing the subthemes found (i.e. participant-provider, work-life balance, behavioural factors, and social environment support), may improve the relationship between participant and HCP, potentially making a participant more willing to adhere to the CLI. Not all of these facilitators and barriers can be directly applied or modified within the design and implementation of CLIs. Nevertheless, they could potentially be addressed and incorporated into the content of CLI sessions.

Comment 4: *There does not seem to have been exploration in the interviews on whether either HCPs and/or CLI participants thought gender barriers could be addressed in CLI design and implementation.*

Response: Thank you for this insightful comment. You are correct that the interviews did not explicitly explore whether healthcare professionals or CLI participants thought that gender-related barriers could be addressed through the design or implementation of the CLI. This was not the primary focus of our study, which aimed to identify gender-related facilitators and barriers rather than to evaluate whether they could be addressed in the CLI design and implementation. However, we agree that this is an important question, as understanding whether healthcare professionals and CLI participants perceive certain facilitators and barriers addressable could inform the development of more targeted interventions. We have therefore acknowledged this gap in our manuscript as a direction for future research.

Action: The following has been added:
Page 22, lines 461-463:

> Whether these gender-related facilitators and barriers in CLIs can be addressed in the design or implementation of CLIs needs to be investigated in future research.

Reviewer: 2
Dr. Hong Ji, Nursing department, The First Affiliated Hospital, Shandong First Medical University

Comments to the Author:

Comment 1: *The Introduction section lacks sufficient logical coherence.*

Response: Thank you for your comment. To improve the logical coherence of the introduction, we have revised the introduction in several places. We have improved the transitions between sentences and paragraphs to improve the overall flow.

Action: The following paragraphs has been revised:
Page 4, lines 80-123:

> Combined lifestyle interventions (CLIs) have shown promise for individuals with knee OA who are overweight and obese.[6] These interventions integrate physical activity, healthy nutrition and behavioural change strategies to support body weight loss and a healthier lifestyle.[7] However, their effectiveness depends not only on clinical outcomes, but also on successful referral and implementation by healthcare professionals (HCPs), as well as, acceptance and adherence by individuals with knee OA. In primary care, HCPs such as general practitioners (GPs) and physical therapists recognise the importance of lifestyle interventions, but reported practical and organisational challenges.[8 9] Furthermore, people's individual circumstances, including gender-related social roles, responsibilities and expectations, determine whether a programme meets their needs and whether they can benefit from it.[10]
>
> Gender-related factors may influence the implementation, uptake, and adherence to CLIs in individuals with knee OA and overweight or obesity.[11] HCPs may have unconscious biases regarding sex and gender,[12] which can influence healthcare given to patients.[13 14] Hence, sex and gender analyses can help to reveal such inequalities and develop strategies to promote more equitable healthcare.[11-13] It is therefore essential to evaluate how HCPs perceive and interact with people of different genders,[15] as these may influence referral patterns, engagement of individuals, and the likelihood of adherence to a CLI. Current insights into this gender-related interaction in the CLI is limited, while understanding it can strengthen the patient-HCP relationship, supporting accessibility and long-term adherence to a CLI.
>
> Although the CLI seems to meet the needs of individuals with overweight and obesity,[16] men and women might differ in their preferences and motivations for a healthy lifestyle. Previous research has shown sex differences in motivations, with men more likely to be motivated by performance and fitness goals, while women are more likely to be motivated by weight management and improving well-being and health.[17] Additionally, men often perceive dieting as feminine.[18 19] Although both physical activity and healthy nutrition are integrated in CLIs, in

practice the emphasis seems to be more on healthy nutrition. This imbalance is reflected in the underrepresentation of men in lifestyle trials and the fact that approximately 70% of CLI participants in the Netherlands are women.[20 21] Difference in preferences, motivations, perceptions, and experiences in the CLI can all influence both participation in and adherence to a CLI. However, the focus has mainly been on the content of lifestyle interventions, while differences in individual circumstances, motivations and communication preferences need to be further investigated.

Comment 2: *The description of the credibility test in qualitative analysis is not detailed enough. It fails to explain the specific process, judgment criteria, and dispute resolution mechanism of the consistency test.*

Response: Thank you for this helpful comment. We have expanded the data analysis section in the methods to clarify the criteria used to assess coder agreement, how the themes and subthemes were created and linked to the domains, and we added the credibility, dependability and confirmability description.

Action: The following has been changed and added:
Page 8, lines 222-243:

> Following a discussion with a third researcher (DS) about the coding trees, the coding trees were revised, and another interview transcript was coded using the revised coding trees. After 75% inter-rater reliability was achieved between two researchers (NJ and PG), based on code definition and overlap in themes, one researcher (PG) coded the remaining transcripts.
>
> For the purpose of this study, we made separate coding trees for each gender identity. If demographic data on self-reported gender identity was missing, we assumed the gender identity of participants on the unspecified question "What is your sex/gender" at the baseline of the LITE study. Themes and subthemes were created based on related codes, and discrepancies were resolved through consensus meeting with the research team (CH, NJ, PG, DS, PB and MvM). For HCPs, we have only focused on the outer setting domain of CFIR, specifically on the influence of gender. For participants, we focused on the domains of outer setting, inner setting, and individuals from CFIR. The themes and subthemes were linked to the above-mentioned domains. Quotes presented for each subtheme are translated from Dutch to English.
>
> Credibility was ensured through triangulation, as we involved both HCPs and CLI participants to capture diverse perspectives. Dependability was supported by a systematic coding process and assessment of inter-rater reliability. Coding decisions and thematic interpretations were refined through discussions within the researcher team. Confirmability was ensured by linking the themes and subthemes to the CFIR framework. The quotes further demonstrate that the findings are supported by the data.

Comment 3: *The classification system needs further optimization and adjustment. The logic of the affiliation of some sub-topics is unreasonable. For example, some content related to "Social Support" is scattered across different themes, and the classification of some subdivisions related to "Motivation" is not clear enough.*

Response: Thank you for this helpful comment. We appreciate your critical perspective on the thematic classification, particularly regarding the subthemes 'Social support' and 'Motivation'. Although the structure was developed through a systematic coding process and the themes and subthemes were generally coherent and well-supported by the data, we agree that some subthemes could be further refined for clarity. We have therefore revised the classification of subthemes as follows:

- Renamed the subtheme "Social support" to "Social environment support", because the data in this subtheme relates to support from the participants' environment and not to support from healthcare professionals, which is already included in the subtheme "Participant-healthcare professional relationship".
- Changed the subtheme "Motivation" to "Behavioural factors", because the underlying content is more related to factors that influence behaviour and participation in the CLI.
- Changed the subtheme "Communication" to "Participant-healthcare professional relationship", which better reflects the content of our findings.

Action: The following has been changed:
Page 11, lines 284-286:

Table 2 – CFIR domain, theme, and subthemes for the implementation of the CLI

CFIR domain and theme	Subthemes	Definition
**Outer setting: Influence of gender**	Participant-HCP relationship	The trust, communication and interaction between participant and HCP in the CLI.
	Work-life balance	Balance between professional and personal life to enable participation in the CLI.
	Behavioural factors	Factors that influence behaviour in the CLI.
	Social environment support	Assistance and comfort from friends, family, and colleagues to participate in the CLI.

Note: CFIR = consolidated framework for implementation research, HCP = healthcare professional, CLI = combined lifestyle intervention.

Comment 4: *The Discussion section is underdeveloped and lacks sufficient depth. The interpretation of research results is superficial, failing to fully conduct comparative analysis with existing relevant studies. The exploration of underlying mechanisms such as gender culture and social structure behind the results is insufficient, and the elaboration on the clinical transformation value of the results is not specific enough.*

Response: Thank you for your comment. We believe that the discussion section already provided a solid and well-balanced interpretation of the findings. Nevertheless, we have further strengthened it by adding literature on the gap between intention and behaviour and on gender differences, which appear inconclusive in the literature. Some studies report that men are more likely to translate intentions into actions, while other studies report the opposite. We have also expanded the discussion on social support by including additional literature that supports our finding that healthcare providers perceive a lack of social support among women. Finally, the clinical translational value of the results is now more clearly described in the second to last paragraph of the discussion.

Action: The following has been changed and added:
Page 21, lines 409-418:

> HCPs perceived resistance from individuals who identified themselves as man or woman with knee OA and overweight or obesity. Participants, however, noted that although they intended to change their behaviour, they experienced difficulties in doing so. This gap between having the intention to change behaviour and actually changing behaviour is known as the intention-behaviour gap.[34] The evidence for gender differences in the intention-behaviour gap remains inconclusive; some studies report that men are more likely to translate intentions into action, whereas other studies find the opposite pattern.[35] Previous research suggests that perceived social support, intention, planning and self-regulation play key roles in bridging the intention-behaviour gap.[36]

Page 21, lines 419-432:

> Of these factors, social environment support was mentioned by both men and women in our study as a key facilitator in the uptake of healthier eating habits, and participation in exercise and lifestyle interventions. HCPs observed a lack of social support among women, which is consistent with the findings of a previous qualitative study on adherence to a dietary lifestyle intervention in people with metabolic syndrome.[37] However, individuals who identified themselves as woman, did not report a lack of social support. Men mentioned that they only received social support from their partners, while women also mentioned they received support from family and friends. This is in line with previous research showing that men mainly received non-work-related social support from their spouses, while women rely more on relatives and friends.[38] Sources of social support appear to vary among individuals, but in general, social support is associated with positive health outcomes.[39] It is therefore important that HCPs discuss the social support in participants' environments and its implications during the CLI sessions.[40]

Page 22, lines 454-463:

> Furthermore, incorporating gender-related facilitators and barriers or at least addressing the subthemes found in the present study (i.e. participant-HCP relationship, work-life balance, behavioural factors, and social environment support), may improve the relationship between participant and HCP, potentially making a participant more willing to adhere to the CLI. Not all of these facilitators

and barriers are directly applicable to the design and implementation of CLIs, but they could potentially be integrated into CLI sessions. Whether these gender-related facilitators and barriers in CLIs can be addressed in the design or implementation of CLIs needs to be investigated in future implementation research.

Comment 5: *Deepen the reflection on the limitations of the research and provide suggestions for future research improvements.*

Response: Thank you for your comment; we have expanded the discussion section accordingly. First, we have added a limitation regarding the lack of interviews with individuals who did not participate in the CLI. This has been added, following Comment 2 of Reviewer 1; please see the response and action above. Including this group might have revealed additional barriers, for example, regarding the decision not to participate. This could have provided important insights, even though our study focused primarily on uptake and adherence among CLI participants. In addition, we have provided suggestions for future research. Following Comment 4 of Reviewer 1 (see response and action above), we propose to investigate how the findings of this study can be integrated into the practical implementation of the CLI. In addition, we now included a recommendation to investigate gender-specific preferences with regarding different elements of the intervention (e.g. physical activity and nutritional advice) and how these preferences may influence adherence and clinical outcomes across different genders.

Action: The following has been added:
Page 22, lines 472-474:
Therefore, future research should investigate whether incorporating these preferences into a CLI can improve adherence to and effectiveness of a CLI across different genders.

VERSION 2 - REVIEW

Reviewer **2**

Name **Ji, Hong**

Affiliation **Nursing department, The First Affiliated Hospital, Shandong First Medical University**

Date **09-Apr-2026**

COI

I have nothing further to add.
